# Supplementary material for: Genetic and Epigenetic Biomarkers Associated with Early Relapse in Pediatric Acute Lymphoblastic Leukemia: A Focused Bioinformatics Study on DNA-Repair Genes
Source: Biomedicines. 2024 Aug 5;12(8):1766. doi: 10.3390/biomedicines12081766 (PMC11351110; doi:10.3390/biomedicines12081766)
Supplement: Supplementary file 1 [file biomedicines-12-01766-s001.zip › biomedicines-3107212-supplementary/biomedicines-3107212-supplementary/Supplementary Figure 1.pdf]

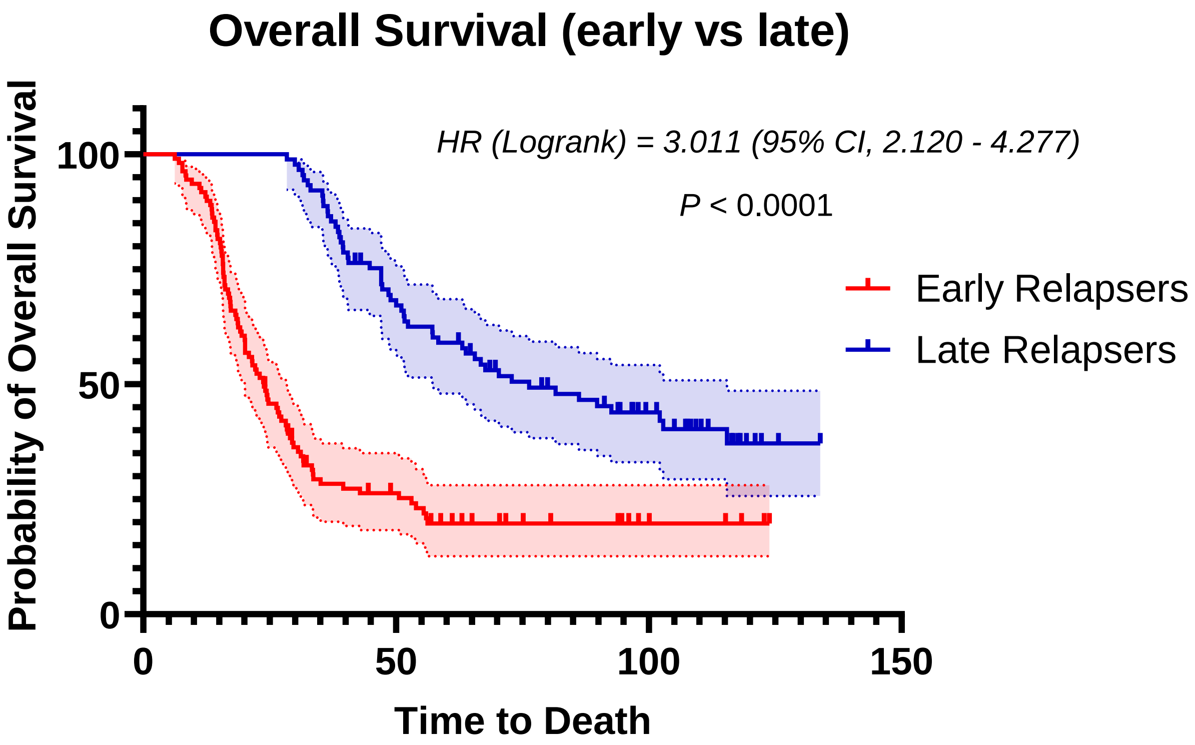

Figure S1. Overall survival curves for early-relapsing patients (red) and late-relapsing patients (blue). The shaded area represents the 95% confidence interval (CI) for each curve. Hazard ratio (HR) and p value were calculated using log-rank test.
